# The effects of sequential attention shifts within visual working memory

**DOI:** 10.3389/fpsyg.2014.00965

**Published:** 2014-09-04

**Authors:** Qi Li, Jun Saiki

**Affiliations:** Graduate School of Human and Environmental Studies, Kyoto UniversityKyoto, Japan

**Keywords:** visual working memory, attention, sequential cueing, retro-cue, selective maintenance

## Abstract

Previous studies have shown conflicting data as to whether it is possible to sequentially shift spatial attention among visual working memory (VWM) representations. The present study investigated this issue by asynchronously presenting attentional cues during the retention interval of a change detection task. In particular, we focused on two types of sequential attention shifts: (1) orienting attention to one location, and then withdrawing attention from it, and (2) switching the focus of attention from one location to another. In Experiment 1, a withdrawal cue was presented after a spatial retro-cue to measure the effect of withdrawing attention. The withdrawal cue significantly reduced the cost of invalid spatial cues, but surprisingly, did not attenuate the benefit of valid spatial cues. This indicates that the withdrawal cue only triggered the activation of facilitative components but not inhibitory components of attention. In Experiment 2, two spatial retro-cues were presented successively to examine the effect of switching the focus of attention. We observed equivalent benefits of the first and second spatial cues, suggesting that participants were able to reorient attention from one location to another within VWM, and the reallocation of attention did not attenuate memory at the first-cued location. In Experiment 3, we found that reducing the validity of the preceding spatial cue did lead to a significant reduction in its benefit. However, performance was still better at first-cued locations than at uncued and neutral locations, indicating that the first cue benefit might have been preserved both partially under automatic control and partially under voluntary control. Our findings revealed new properties of dynamic attentional control in VWM maintenance.

## INTRODUCTION

Visual working memory (VWM) refers to the cognitive ability that allows us to temporarily store a limited amount of visual information and manipulate this information online (Baddeley and Hitch, 1974; Baddeley, 1992; Cohen et al., 1997; Luck and Vogel, 1997; Smith and Jonides, 1999; Fougnie, 2009). There is increasing evidence that VWM closely interacts with visual attention (see Awh and Jonides, 2001 for a review). For example, some studies have found that perceptual representations matching the contents of VWM capture visual attention, indicating that information held in VWM can bias the allocation of attention (Pashler and Shiu, 1999; Downing, 2000; Soto et al., 2005). Other studies have demonstrated that attention can effectively bias the encoding and consolidation processes of VWM (Rensink et al., 1997; Becker et al., 2000; Scholl, 2000; Schmidt et al., 2002; Woodman et al., 2003).

More recently, a growing body of research using a retro-cue paradigm has begun to highlight the role of visual attention in VWM maintenance (Griffin and Nobre, 2003; Landman et al., 2003; Nobre et al., 2004; Matsukura et al., 2007; Makovski et al., 2008; Berryhill et al., 2011; Tanoue and Berryhill, 2012). In a typical retro-cue experiment, an attentional cue indicating a to-be-maintained VWM representation is presented during the retention interval of a change detection task. Previous research has consistently reported enhanced memory for the retrospectively cued representation. Because the retro-cues were presented long after (> 1 s, beyond the range of iconic memory) the memory display offset, the retro-cue effect has been taken as evidence that visual attention can operate on mental representations during VWM maintenance. Moreover, studies directly comparing attentional selection of perceptual and mental representations have revealed considerable behavioral and neural similarities in these two cases (e.g., Nobre et al., 2004; Kuo et al., 2009; Munneke et al., 2010; Katus et al., 2014), indicating that attention works in a similar way on the mental representations as on perceptual representations.

In the perceptual domain, it is generally agreed that one important aspect of efficient information processing is the ability to quickly and frequently reorient attention, and there has been considerable research investigating sequential attention shifts among perceptual representations. One of the most famous studies was carried out by Posner and Cohen (1984). The researchers developed a sequential cueing procedure, which allows observation of attention reorienting. In their experiments, participants detected a visual target that could be presented at one of three possible locations (left, center, and right). Before the target’s onset, participants were first cued to a peripheral location, then after a short time, cued to the central location. Responses to targets were faster at the first-cued location than at the uncued location when cue-target onset asynchrony (CTOA) was short (within about 250 ms). But when CTOA was long (>250 ms), responses to targets were slower at the first-cued location relative to the uncued location. Posner and Cohen (1984) and Posner et al. (1985) suggested that this inhibitory effect (inhibition of return, IOR) reflects a mechanism that encourages perceptual processing of novel items. The IOR effect is robust and has been widely replicated under many different experimental conditions. For example, studies have shown that not only exogenous cues but also endogenous cues can be used to produce IOR (Weger et al., 2008); IOR occurs in both discrimination tasks and detection tasks (Lupiáñez et al., 1997; Pratt and Abrams, 1999); there are both space- and object-based IOR (Tipper et al., 1991; Abrams and Law, 2000); IOR emerges with visual cues as well as auditory cues (Mondor et al., 1998; Mayer et al., 2007).

Although the mechanisms of sequential attention shifts have been extensively studied in the perceptual domain, very few studies have investigated sequential attention shifts within VWM. To the best of our knowledge, only four studies have addressed this issue. All these studies combined Posner et al.’s (1985) sequential cueing procedure with the retro-cue paradigm. Two of them investigated object-based attention (Lepsien and Nobre, 2007; Hollingworth and Maxcey-Richard, 2013), and both reported successful reorientation of attention to VWM representations. For example, in the fMRI study of Lepsien and Nobre (2007), participants were successively presented with two memory items from different object categories (face and scene), and then given a visual cue to direct attention to one object (either the face or scene), and later further cued to redirect attention to the uncued object. The second retro-cue produced behavioral benefits and elicited content-specific activation, giving evidence that object-based attention can be redirected to the previously uncued representation. Hollingworth and Maxcey-Richard (2013, Experiment 3) further demonstrated that the content of VWM can be successively modulated by auditory retro-cues that informed participants whether a previously viewed object was more or less likely to be tested. The consistent results across these two studies strongly indicate a flexible VWM maintenance mechanism that allows resource reallocation via object-based selection. However, with respect to reorienting spatial attention within VWM, research shows conflicting results. In the study of Landman et al. (2003, Experiment 3), after memorizing an array of eight items, participants were cued to attend one location, and in some trials, further cued to reorient attention to another location. The results suggested successful reorientation of spatial attention by showing equivalent change detection performance for sequential and single cueing. The authors therefore concluded that orienting attention to a VWM representation does not render other representations unavailable. However, this conclusion was drawn based on non-significant differences between conditions, and the lack of statistical significance can be interpreted in other ways rather than success in reorienting. For example, Matsukura et al. (2007) pointed out that the equivalent performance for sequential cueing and single cueing can be due to that participants ignored the first cue on sequential cueing trials, or due to a ceiling effect in all conditions. Indeed, Matsukura et al. (2007, Experiment 3, 4) conducted similar experiments, but found significantly worse performance for reorienting (the “double-cue, different-direction” condition) compared with shifting attention only once (the “double-cue, same-direction” condition). Based on these results, Matsukura et al. (2007) suggested that cued representations are protected at the cost of losing other uncued representations. However, their primary conclusions can also be challenged by other interpretations of the results. For example, the worse performance for reorienting may result from participants’ strategies of ignoring the second cue. In Matsukura et al.’s (2007) experiments, there were only a small number of trials requiring reorientation of attention (11% of total), whereas on most trials participants shifted attention only once. This experimental design can lead to preference for the first cue over the second cue. Actually, the authors provided evidence that participants used the first cue by showing validity effects in the “single-cue” condition, but there was no evidence to confirm that participants did not ignore the second cue. Another plausible reason for the low performance in the “double cue, different direction” condition may relate to the number of objects indicated by the spatial cue. In Matsukura et al.’s (2007) experiments, a spatial cue indicated a set of two or three to-be-maintained representations, which could largely increase the difficulty of accessing/retrieving all the cued representations. There is a possibility that participants are capable of reorienting spatial attention when the load of attentional selection is reduced to one.

As reviewed above, studies so far have produced inconsistent results and brought on many unresolved questions about reorienting spatial attention within VWM. The purpose of the present study was to further examine the nature of reorienting spatial attention among mental representations. In particular, we systematically investigated two types of reorienting: (1) orienting attention to one location, and then withdrawing attention from it; and (2) switching the focus of attention from one location to another.

## EXPERIMENT 1

In Experiment 1, we tested whether participants could withdraw attention from a previously attended location and return to the state before the first orientation of attention, by presenting a spatial cue first, and then a withdrawal cue during the retention interval of a change detection task. Two recent studies have demonstrated that participants can use retro-cues to forget a subset of task-irrelevant VWM representations, leading to improved memory for the remained task-relevant items (Williams and Woodman, 2012; Williams et al., 2013). These directed-forgetting studies have highlighted great flexibility in the use of retro-cues. Thus, if retrospectively attending one location did not render representations at the uncued locations unavailable as Landman et al. (2003) proposed, we would expect to see evidence that participants used the withdrawal cue to recover uncued representations. In contrast, if spatially retro-cued representations were protected at the cost of losing other uncued representations as Matsukura et al. (2007) proposed, participants should not be able to take advantage of the withdraw cue to recover the initially uncued representations.

### MATERIALS AND METHODS

#### Participants

Sixteen students from Kyoto University (aged 18–20 years, four females) participated in the experiment after giving informed consent. All participants reported normal color vision and normal or corrected-to-normal visual acuity. The experiment protocol was approved by the Institutional Review Board of Kyoto University.

#### Apparatus

The experiment was conducted in a darkened testing room. Participants sat 57 cm away from a 21-inch CRT monitor (75 Hz refresh rate; 1024 × 768 resolution), with their head immobilized by a chin rest, forehead rest, and temple stabilizers. Visual stimuli were generated using Psychophysics Toolbox (Brainard, 1997; Pelli, 1997) implemented in Matlab.

#### Stimuli

Stimuli were presented on a gray background (21.6 cd/m^2^). The memory display consisted of four colored crosses (red, blue, green, and yellow). Each cross subtended 0.78^∘^ in height and width. The crosses were centered 3.82^∘^ above, below, to the left, and to the right of the central fixation point. In the probe display, a colored cross (red, blue, green, or yellow) appeared at one of the four locations occupied by the memory array stimuli. The cue display consisted of either a neutral cue (“+,” 0.78 height and width), a spatial cue (arrow, 0.78 height and width), or a withdrawal cue (“⊖,” 0.78^∘^ height and width).

#### Design and procedure

**FIGURE 1:**
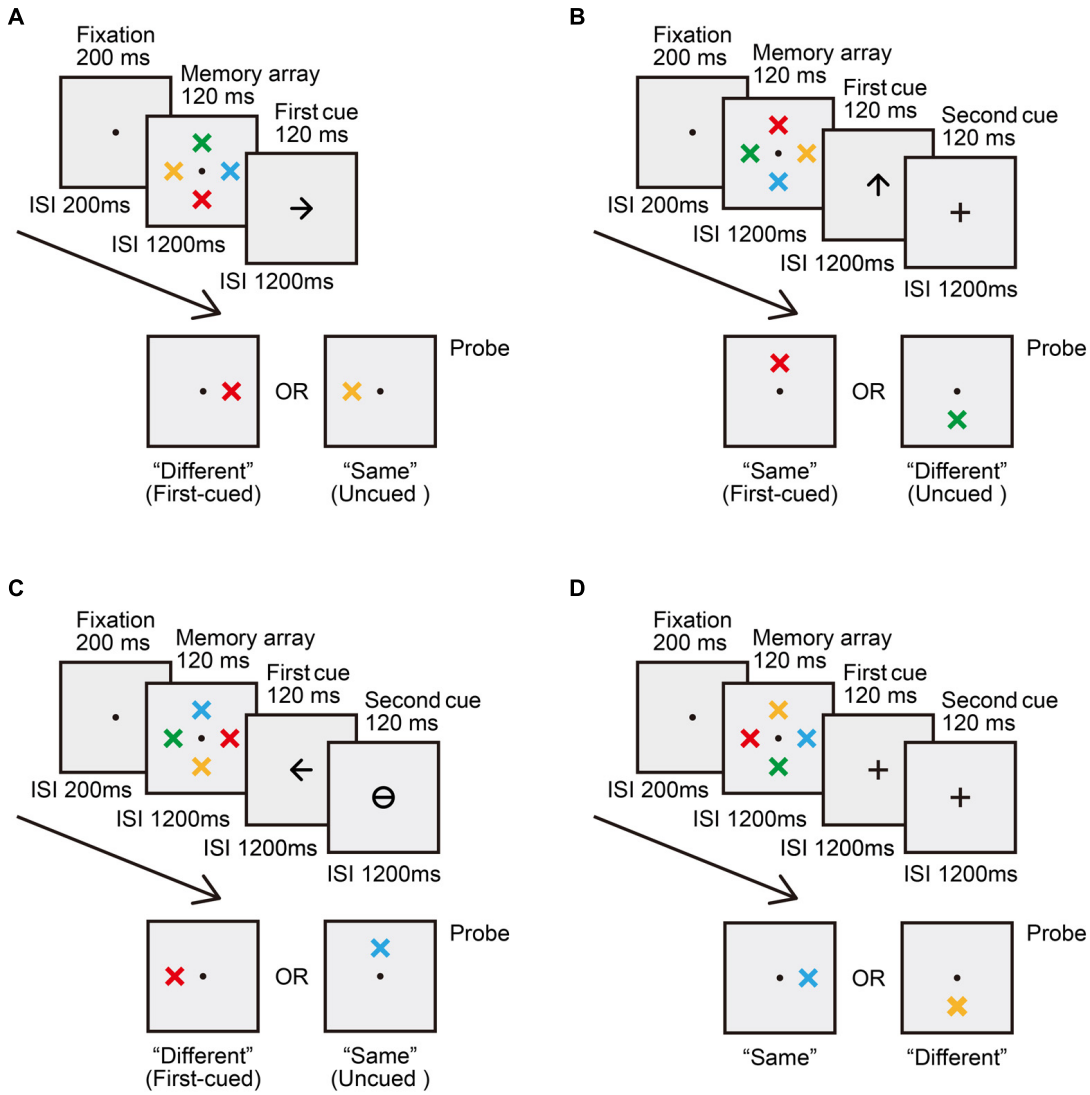
**Example trials for the four cue type conditions in Experiment 1. (A)** The “single shift + early probe” (SE) condition. **(B)** The “single shift + late probe” (SL) condition. **(C)** The withdrawal condition. **(D)** The neutral condition.

**FIGURE 2:**
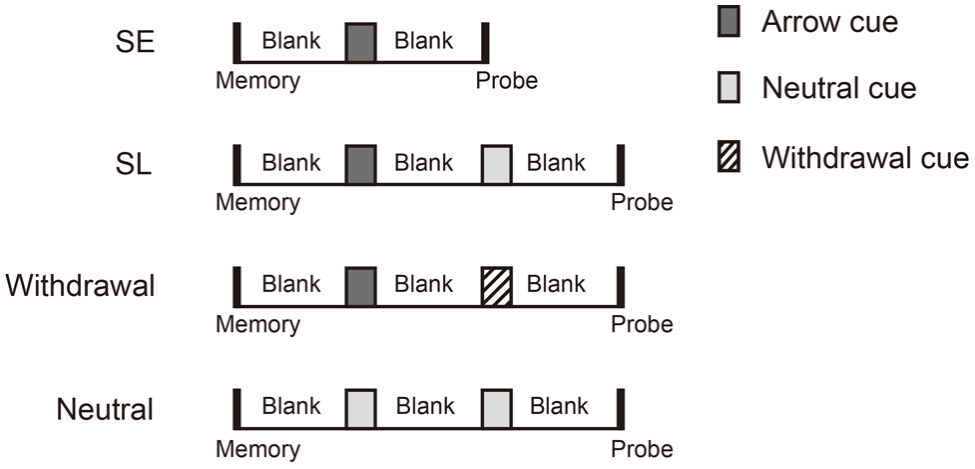
**Schematic picture of the stimulus sequence between memory array offset and probe onset for each cue type condition in Experiment 1**.

There were four cue type conditions: (1) the “single shift + early probe” (SE) condition, (2) the “single shift + late probe” (SL) condition, (3) the withdrawal condition, and (4) the neutral condition. These conditions were randomly mixed. **Figure 1** shows the sequence of events for a typical trial in each condition. Trials were self-initiated, and began with the onset of a central fixation point. In the SE condition (**Figure 1A**), the fixation point appeared for 200 ms, followed by a 200-ms blank interstimulus interval (ISI), followed by a 120-ms memory array. After a 1200-ms blank ISI, the first cue display consisting of an arrow cue appeared for 120 ms. After another 1200-ms blank ISI, the probe display consisting of one colored cross was presented until response. In the SL condition (**Figure 1B**), trials also contained a 200-ms fixation point, a 200-ms blank screen, a 120-ms memory array, a 1200-ms blank screen, and a 120-ms first cue display. But after the 1200-ms blank ISI following the first cue display, a second cue display was presented for 120 ms. The second cue display consisted of a neutral cue. This was followed by another 1200-ms blank ISI. After this ISI, a probe display was presented until response. Trials in the withdrawal condition contained the same sequence of events as those in the SL condition, except that in the second cue display, a withdrawal cue instructing participants to withdraw attention from the first-cued item was presented (**Figure 1C**). Trials in the neutral condition also consisted of a fixation, a memory array, a first cue, a second cue and a probe display, but both the first and second cues were neutral cues (**Figure 1D**). **Figure 2** shows the differences in stimulus sequence between memory array offset and probe onset in the four cue type conditions. Participants’ task was to memorize the color and location of each item in the array and decide whether the probe had the same color as the item presented at the corresponding location in the memory array. Accuracy was emphasized rather than response speed.

The participants received both oral and written instructions, and were given printed images describing the task, stimuli, and experimental conditions. Specifically, they were instructed that (1) a spatial cue required them to orient attention to the location indicated by the cue; (2) a withdrawal cue required them to withdraw attention from the attended location and return to the state before the first orienting, more specifically, return to the state of maintaining all four representations equivalently; and (3) a neutral cue required them to keep the current state, that is, the neutral cues following the memory array required them to keep maintaining all four items in the array (the neutral condition), and the neutral cue following the spatial cue required them to keep attending the spatially cued representation (the SL condition).

Each participant completed 12 blocks of 20 trials, resulting in 240 trials in total (48 SE, 48 SL, 96 withdrawal, 48 neutral). Whenever a spatial cue was presented, the probe would appear at the cued location 50% of the time (first-cued trials; in this case, the spatial cue was a valid cue), and appear at any one of the three uncued locations approximately 17% of the time each location (uncued trials; in this case, the spatial cue was an invalid cue). The probability that the color of the probe was the same as or different from the item at the corresponding location in the memory array was equal (50%). On *different* trials, the color of the probe was randomly selected from the other three colors in the array, with the constraint that on *uncued-different* trials, the probe could not be the color from the cued location. All trial types were presented in a random order throughout the experiment. The assignment of “same” and “different” values to response keys was counterbalanced across participants. Before the experimental blocks, participants performed 1–2 practice blocks to ensure they understood the task.

#### Data analysis

Participants’ performance was measured with the sensitivity index *d*′. The *d*′ scores were calculated as: *d*′ = *z*(hit rate)–*z*(false alarm rate), where the hit rate was the proportion of *different* trials to which participants responded “different,” and the false alarm rate was the proportion of *same* trials to which participants responded “different.”

### RESULTS AND DISCUSSION

**FIGURE 3:**
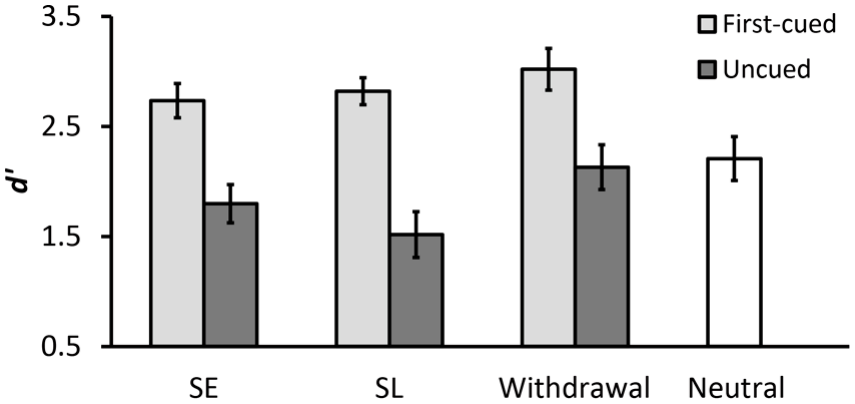
**Mean *d*′ for each cue type condition in Experiment 1.** Error bars represent standard errors.

**Table 1** summarizes the results of mean hit rates and mean false alarm rates. **Figure 3** shows the results of *d*′. We compared the cueing effects in SE, SL, and withdrawal conditions using a two-way repeated-measures analysis of variance (ANOVA) with cue type (SE, SL, withdrawal) and validity (first-cued, uncued) as within-subjects factors. There was a significant main effect of validity [*F*(1,15) = 42.21, *p* < .001, $\eta_{p}^{2}$ = .74], indicating higher *d*′ for first-cued trials compared to uncued trials. There was also a significant main effect of cue type [*F*(2,30) = 6.31, *p* = .005, $\eta_{p}^{2}$ = .30], indicating differences in *d*′ between the three cue type conditions. *Post hoc* analyses revealed that the withdrawal condition had significantly higher *d*′ than both SE and SL conditions [withdrawal vs. SE: *t*(15) = 2.48, *p* = .026; withdrawal vs. SL: *t*(15) = 3.16, *p* = .020]. In addition, the interaction between cue type and validity approached significance [*F*(2,30) = 2.61, *p* = .090, $\eta_{p}^{2}$ = .15]. Further analyses of the interaction revealed a significant simple main effect of validity in all three cue type conditions [SE: *F*(1,15) = 21.37, *p* < .001, $\eta_{p}^{2}$ = .59; SL: *F*(1,15) = 39.22, *p* < .001, $\eta_{p}^{2}$ = .72; withdrawal: *F*(1,15) = 24.49, *p* < .001, $\eta_{p}^{2}$ = .62], indicating that *d*′ was significantly higher on first-cued trials versus uncued trials in each cue type condition. Moreover, there was a significant simple main effect of cue type for uncued trials [*F*(2,30) = 7.04, *p* = .003, $\eta_{p}^{2}$ = .32], but no such an effect for first-cued trials (*p* > .15). This indicates that *d*′ in SE, SL, and withdrawal conditions was equivalent when the first-cued location was tested, but when an uncued location was tested, *d*′ differed significantly among the three cue type conditions. Specifically, on uncued trials, the withdrawal condition had significantly higher *d*′ than the other two conditions [withdrawal vs. SE: *t*(15) = 2.24, *p* = .041; withdrawal vs. SL: *t*(15) = 4.12, *p* = .003]. It is important to note that the SL and withdrawal conditions only differed in whether a spatial cue was followed by a neutral cue or by a withdrawal cue. Thus, the observed significant worse performance on uncued SL trials compared to uncued withdrawal trials suggests that the disadvantages of invalid spatial cues were effectively reduced by the withdrawal cue.

**Table 1:** Mean hit rates and mean false alarm rates for each condition in Experiment 1 (Standard errors are shown in parentheses).

	SE		SL		Withdrawal		Neutral
	Cued	Uncued	Cued	Uncued	Cued	Uncued	
Hit (%)	89.6	85.9	93.8	82.6	93.2	89.5	90.6
	(2.4)	(3.2)	(1.3)	(2.8)	(1.8)	(2.5)	(2.3)
False Alarm (%)	10.7	29.7	12.5	34.1	11.1	27.3	25.3
	(2.9)	(4.9)	(2.0)	(5.8)	(2.4)	(4.7)	(4.1)

We also assessed the spatial cueing benefit and cost in the withdrawal condition by comparing the withdrawal and neutral conditions using an ANOVA with validity (first-cued, uncued, neutral) as the within-subjects factor. There was a significant main effect of validity [*F*(2,30) = 14.75, *p* < .001, $\eta_{p}^{2}$ = .50]. *Post hoc* analyses revealed that *d*′ was significantly higher on first-cued trials compared to uncued and neutral trials [first-cued vs. uncued: *t*(15) = 4.95, *p* < .001; first-cued vs. neutral: *t*(15) = 4.13, *p* < .001], but the difference between the uncued and neutral condition did not approach significance (*p* > .64), indicating that there was only a benefit of valid spatial cueing but no cost of invalid spatial cueing relative to the neutral baseline when the withdrawal cue was presented.

Our results show that participants were able to use the withdrawal cue to improve VWM performance, and therefore support the hypothesis that uncued representations are available for reorientation of attention. The SE condition was included to provide manipulation checks on whether participants ignored the first spatial cue. The strong validity effect observed in this condition provides clear evidence that the selection of a spatially cued representation was accomplished before the onset of the second cue. Thus, the reduced cost in the withdrawal condition should reflect modulation of VWM contents by withdrawing or reallocating attention rather than merely ignoring the preceding spatial cue. In addition, the validity effects in the two single-shift conditions (SE, SL) replicated the findings of previous retro-cue research, suggesting that the withdrawal cueing effect was not due to some peculiarities of the current experiment.

An unexpected observation is that the benefit of valid spatial cueing was preserved in the withdrawal condition. This indicates that it is possible to recover representations at the uncued locations without attenuating representation at the previously cued location. Note that the spatial cue was informative even on withdrawal trials. This could lead to participants’ voluntary control over the suppression of the previously attended location.

## EXPERIMENT 2

In Experiment 2, we investigated the processes of reorienting attention from one location to another during VWM maintenance by presenting a second spatial cue after the first one. If participants were able to reorient attention, there should be a second spatial cue benefit. In addition, the results of Experiment 1 suggest that the first cue benefit can be preserved after reorienting. The current experiment further examined whether this effect was robust in different type of reorienting.

### METHOD

**FIGURE 4:**
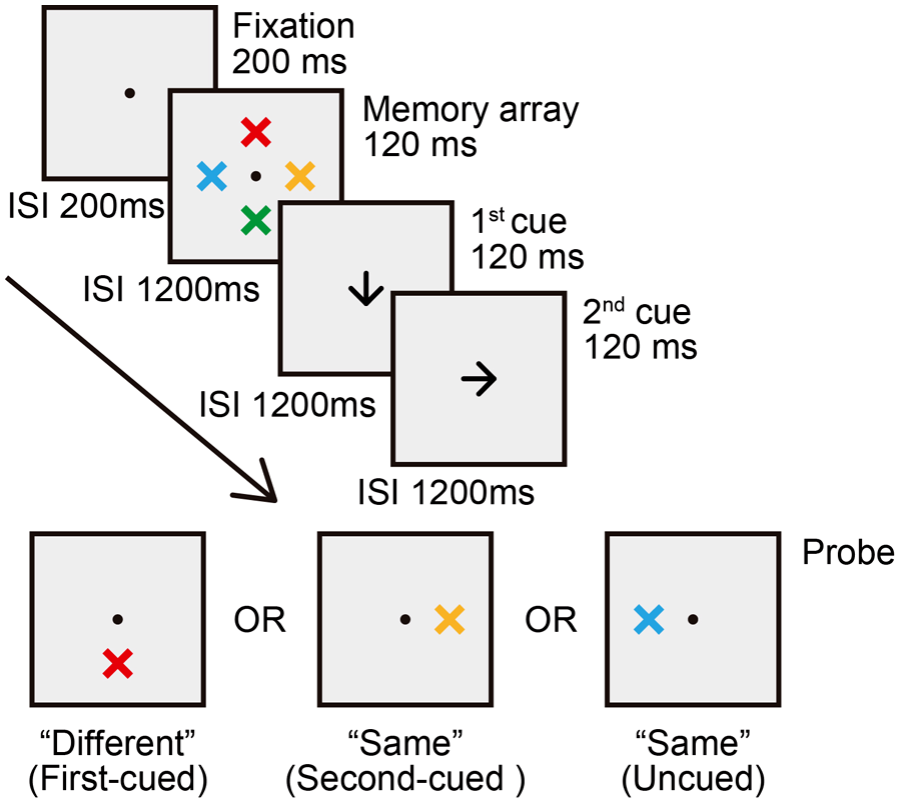
**Trial sequence for the “double spatial cue” (DS) condition in Experiment 2 and 3**.

The experimental method of this experiment was identical to that used in Experiment 1 with the following exceptions. Sixteen new participants (aged 18–23 years, four females) took part in this experiment. There were four cue type conditions: (1) the SE condition, (2) the SL condition, (3) the “double spatial cue” (DS) condition, and (4) the neutral condition. SE, SL, and neutral conditions were exactly the same as those in Experiment 1. In the DS condition, the first spatial cue was followed by a second spatial cue, instructing participants to reorient attention to a new location (see **Figure 4**). The direction of the first spatial cue was randomly selected from the four possible directions (up, down, left, and right). The direction of the second spatial cue was randomly selected from the other three unused directions. There were 48 SE, 48 SL, 144 DS, and 48 neutral trials. Similar to Experiment 1, in the two single-shift conditions, the probe appeared at the cued location with 50% probability (first-cued trials; i.e., valid first cue), and at any one of the other uncued locations with approximately 17% probability each location (uncued trials; i.e., invalid first cue). In the DS condition, the probe appeared at the first-cued location with a probability of approximately 33% (first-cued trials; i.e., valid first cue), at the second-cued location with an equal probability of approximately 33% (second-cued trials; i.e., valid second cue), and at either one of the two uncued locations with approximately 17% probability each location (uncued trials; i.e., invalid both first and second cues).

### RESULTS AND DISCUSSION

**FIGURE 5:**
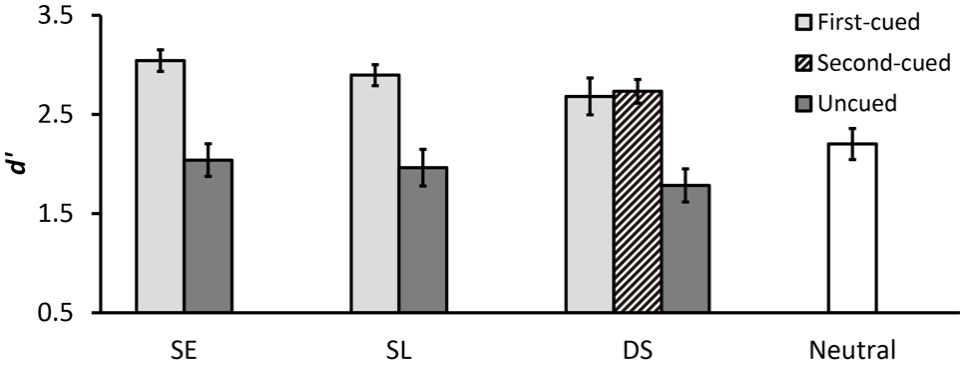
**Mean *d*′ for each cue type condition in Experiment 2.** Error bars represent standard errors.

**Table 2** summarizes the results of mean hit rates and mean false alarm rates. **Figure 5** shows the results of mean *d*′. The validity effect of the first cue was confirmed by a two-way ANOVA with cue type (SE, SL, DS) and validity (first-cued, uncued) as within-subjects factors. There was a significant main effect of validity [*F*(1,15) = 53.38, *p* < .001, $\eta_{p}^{2}$ = .78], indicating higher *d*′ for first-cued trials compared to uncued trials. There was no interaction between factors (*p*> .91), and therefore the pattern of higher *d*′ for first-cued versus uncued trials was equivalent for SE, SL, and DS conditions.

**Table 2:** Mean hit rates and mean false alarm rates for each condition in Experiment 2 (Standard errors are shown in parentheses).

	SE		SL			DS		Neutral
	Cued	Uncued	Cued	Uncued	1st cued	2nd cued	Uncued	
Hit (%)	94.0	89.8	93.5	88.5	93.4	93.4	87.0	90.8
	(.8)	(2.0)	(.8)	(2.2)	(1.1)	(1.0)	(2.8)	(1.8)
False alarm (%)	8.6	27.6	10.2	28.1	16.7	13.5	32.3	25.0
	(1.7)	(3.9)	(1.6)	(4.6)	(2.6)	(2.1)	(4.2)	(3.9)

After confirming that participants have used the first cue to orient attention, we set out to compare the DS condition with the neutral condition to assess the benefit and cost of sequential spatial cueing. An ANOVA with the within-subjects factor of validity (first-cued, second-cued, uncued, neutral) yileded a significant main effect of validity [*F*(3,45) = 12.88, *p* < .001, $\eta_{p}^{2}$ = .46]. *Post hoc* analyses revealed that both first- and second-cued trials had significantly higher *d*′ than neutral trials [first-cued vs. neutral: *t*(15) = 2.72, *p* = .009; second-cued vs. neutral: *t*(15) = 3.01, *p* = .004], indicating that change detection performance was enhanced when a probe appeared at the first- or second-cued location. *d*′ was significantly lower on uncued trials than on neutral trials [*t*(15) = 2.37, *p* = .022], revealing a cost when an uncued location was tested. In addition, *d*′ on first- and second-cued trials did not differ significantly (*p* > .77), indicating that the first and second spatial cues produced equivalent benefits in the DS condition.

The second spatial cue benefit indicates that representations outside the current focus of attention can be reselected with the help of a second spatial cue. Interestingly, like experiment 1, the reorientation of attention did not lead to attenuation of the first cue benefit. The preserved first cue benefit can be explained by assuming that once the information held in VWM has been facilitated by focused attention, sustained allocation of focused attention is not necessary to maintain this facilitated information. Another explanation is based on cue validity. In this experiment, the probe appeared at the first- and second-cued locations with the same probability in the DS condition (approximately 33%, higher than an uncued location). Participants may have voluntarily maintained both the first- and second-cued representations. If this is the case, reducing first cue validity should result in a corresponding reduction of the first cue benefit. Experiment 3 examined this issue by lowering the first cue validity.

## EXPERIMENT 3

The observation of a large benefit of the preceding spatial cue in our first two experiments may reflect that facilitated VWM information can be maintained without sustained focused attention. However, it is also possible that the first cue benefit is determined merely by cue validity. In this experiment, we tested the validity account by reducing the validity of the first spatial cue in the DS condition. Specifically, once the participant viewed a second spatial cue, the probe would appear at the second-cued location with a probability of 50%, at any one of the other three locations with a probability of approximately 17% each location. Thus, when a second spatial cue appeared, the first spatial cue became non-informative of the probe’s location. A pure validity account predicts best performance at the second-cued location, but equivalent performance at the first-cued and uncued locations. On the other hand, if performance was still better at the first-cued location than at the uncued locations, the first cue advantages should not solely depend on cue validity.

### METHOD

The experimental method of this experiment was identical to that used in Experiment 2 except that in the DS condition, the probe appeared at the second-cued location with a probability of 50%, but appeared at the first-cued or an uncued location with a probability of approximately 17%. Eighteen new participants (aged 19–26 years, 10 females) took part in this experiment.

### RESULTS AND DISCUSSION

**FIGURE 6:**
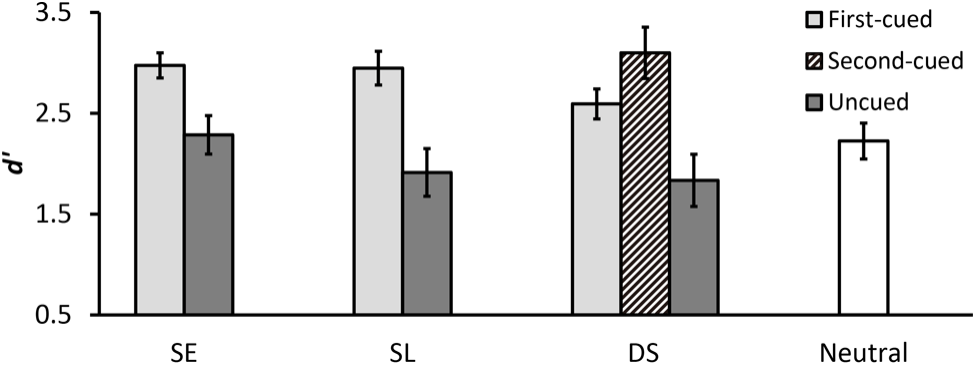
**Mean *d*′ for each cue type condition in Experiment 3.** Error bars represent standard errors.

**Table 3** summarizes the results of mean hit rates and mean false alarm rates. **Figure 6** shows the results of mean *d*′. Similar to Experiment 2, a two-way ANOVA with cue type (SE, SL, DS) and validity (first-cued, uncued) as within-subjects factors yielded a significant main effect of validity [*F*(1,17) = 39.33, *p* < .001, $\eta_{p}^{2}$ = .70], indicating higher *d*′ for first-cued trials compared to uncued trials. There was no interaction between factors (*p*> .31), suggesting that the pattern of higher *d*′ for first-cued versus uncued trials was equivalent for SE, SL, and DS conditions. These results provide evidence that participants did not ignore the first spatial cue.

**Table 3:** Mean hit rates and mean false alarm rates for each condition in Experiment 3 (Standard errors are shown in parentheses).

	SE		SL			DS		Neutral
	Cued	Uncued	Cued	Uncued	1st cued	2nd cued	Uncued	
Hit (%)	91.0	82.9	93.3	85.2	91.9	92.4	82.5	89.9
	(1.9)	(3.9)	(1.0)	(3.0)	(1.2)	(1.4)	(2.9)	(2.0)
False alarm (%)	11.6	19.2	11.6	26.4	17.1	12.7	26.5	26.0
	(3.1)	(3.6)	(2.4)	(4.4)	(3.5)	(3.0)	(3.9)	(3.8)

Next, we focused on comparing the DS condition with the neutral condition to examine the benefit and cost of sequential spatial cueing. An ANOVA with the within-subjects factor of validity (first-cued, second-cued, uncued, neutral) yielded a significant main effect of validity [*F*(3,51) = 17.58, *p* < .001, $\eta_{p}^{2}$ = .51]. *Post hoc* analyses revealed a significant second cue benefit by showing highest *d*′ on second-cued trials [second-cued vs. first-cued: *t*(17) = 2.64, *p* = .011; second-cued vs. uncued: *t*(17) = 6.85, *p* < .001; second-cued vs. neutral: *t*(17) = 4.98, *p* < .001]. In addition, *d*′ was still significantly higher on first-cued trials than on uncued and neutral trials [first-cued vs. uncued: *t*(17) = 4.21, *p* < .001; first-cued vs. neutral: *t*(17) = 2.35, *p* = .023], indicating significant first cue advantages.

In this experiment, performance was significantly better at the second-cued location than at the first-cued location, suggesting that the magnitude of the first cue benefit can be modulated by cue validity. However, performance was still better on first-cued trials than on uncued and neutral trials. This is inconsistent with the pure validity account. It seems that the first cue benefit has been preserved partially under automatic control and partially under voluntary control.

## GENERAL DISCUSSION

The present study used the sequential retro-cueing procedure to investigate the processes of reorienting spatial attention within VWM. In all three experiments, participants’ performance was significantly modulated by both the first and second attentional cues. In Experiment 1, the second cue instructing participants to withdraw attention from the first-cued location significantly reduced the cost of invalid first cues. But the benefit of valid first cues was not attenuated by the withdrawal cue. In Experiments 2 and 3, two spatial cues indicated different locations were successively presented during the retention interval. In Experiment 2, both the first and second spatial cues were informative, whereas in Experiment 3, only the second spatial cue was informative. In both experiments, the first and second cues produced significant behavioral benefits. In Experiment 2, the first and second cues facilitated change detection performance to an equivalent extent. However, in Experiment 3, the second cue facilitated performance to a greater extent than the first cue did. These results allow new insights into the dynamic modulation of VWM maintenance by visual attention.

### IS IT POSSIBLE TO SUCCESSIVELY SHIFT ATTENTION WITHIN VWM?

Visual attention is subject to severe capacity limitations (Pylyshyn and Storm, 1988; Yantis, 1992; Scholl et al., 2001; Cavanagh and Alvarez, 2005; Fougnie and Marois, 2006). The ability of rapidly reorient attention is important to succeed in a wide range of cognitive tasks. Recent retro-cue research has demonstrated that participants can shift attention to mental representations during VWM maintenance (e.g., Griffin and Nobre, 2003). Is it possible to sequentially shift attention among VWM representations? Three experiments were conducted to answer this question. In all experiments, we found reliable sequential retro-cueing effects, suggesting that the answer is “yes.” In Experiment 1, presenting the withdrawal cue after a spatial retro-cue significantly decreased the cost of preceding invalid spatial cues. In Experiments 2 and 3, a second spatial retro-cue significantly enhanced performance at that location. In addition, the validity effects observed in the SE condition in each experiment rule out the possibility that the second cue effects are simply due to ignoring the first cue or postponing orienting attention to the first-cued location. Our findings support the ideas of Landman et al. (2003), who suggested that attending one representation in VWM does not render the other uncued representations unavailable. Furthermore, our results are consistent with previous research demonstrating successful object-based reorienting within VWM (Lepsien and Nobre, 2007), and therefore have important implications for a general model of attention reorienting. However, as the present study only investigated reorienting among four simple VWM representations, the pronounced second cue effects may not be generalized and transferred to other situations using complex visual stimuli or large set sizes. Further experimental investigations are needed to clarify whether the efficiency of attention reorienting within VWM depends on memory load.

### THE EFFECT OF THE FIRST SPATIAL CUE IN SEQUENTIAL RETRO-CUEING

One unanticipated finding was that the facilitative effect conferred by the first cue remained after the reorientation of attention. In Experiment 1, the second cue that instructed participants to withdraw attention from the first-cued location significantly reduced the cost of invalid first cues, but did not attenuate the benefit of valid first cues. In Experiment 2, the second spatial cue that instructed participants to switch the focus of attention from the first-cued location to the second-cued location did lead to facilitation at the second-cued location, but the first cue prior to the second cue facilitated performance to the same extent as the second cue did. In Experiment 3, different from Experiments 1 and 2, the preceding spatial cue was completely non-informative when two spatial cues were sequentially presented. This resulted in significantly worse performance at the first-cued location compared to the second-cued location in the DS condition, indicating that cue validity can effectively modulate the benefit of the preceding spatial cues. Thus, the big benefit of the preceding cues in Experiments 1 and 2 may derive from voluntary control. That is to say, participants in our first two experiments might notice that the first cue was informative even when a second cue was given, and therefore actively and consciously preserved the first-cued representation. Furthermore, Experiment 3 revealed that the magnitude of the preceding spatial cue benefit significantly decreased with reduced cue validity, providing evidence for the voluntary control account. However, even in Experiment 3, performance was significantly better at the first-cued location than at uncued and neutral locations. The preserved first cue benefit in Experiment 3 cannot be fully explained by the voluntary control account. What is the underlying mechanism? One possibility is that facilitated information due to spatial retro-cues can be maintained without sustained focused attention. In our experiments, successful performance required correct memory for color-location bindings. Memory for the color-location binding at the cued location may be facilitated by spatial retro-cues which caused deployment of focused attention. If the facilitated binding information of the initially cued representation can be maintained without sustained focused attention, the benefit of the preceding spatial cue would be preserved in spite of attention reorienting. Several recent studies have suggested that feature bindings of integrated VWM representations can be maintained at the absence of sustained focused attention (Johnson et al., 2008; Delvenne et al., 2010). Our results seem to be consistent with this view by showing that the first retro-cue can facilitate binding task performance even when attention has been reoriented to other representations.

### PROTECTION AND PRIORITIZATION

Two major hypotheses, the protection and prioritization hypotheses, have been proposed to account for the retro-cue benefit (e.g., Griffin and Nobre, 2003; Matsukura et al., 2007). The protection hypothesis supposes that retro-cues change the quality of representations at cued and uncued locations. On the other hand, the prioritization hypothesis proposes that retro-cues change the priority of representations at cued and uncued location. In the case of sequential retro-cueing, the protection hypothesis will predict a failure in using a second attentional cue, whereas the prioritization hypothesis will predict facilitative effect of a second attentional cue (Matsukura et al., 2007). Our results seem to be more consistent with the prioritization hypothesis by consistently showing facilitated effects of the second attentional cues. However, the prioritization hypothesis cannot fully account for the remained first cue benefits after attention reorienting. For example, in Experiment 1, if the priority given to the first-cued representation was canceled by the second withdrawal cue, performance at the first-cued location should not differ from the other locations. However, we observed significant better performance at the first-cued location compared to uncued locations. It seems that the current findings of facilitative effects for both the first and second cues rely not only on the prioritization mechanism, but also on the protection mechanism. The protection and prioritization hypotheses have been distinguished as two different mechanisms. Our results suggest that these hypotheses are not necessarily mutually exclusive, and combination of them may further refine performance predictions in various retro-cued memory tasks.

### COMPARISON WITH PREVIOUS SEQUENTIAL RETRO-CUEING STUDIES

Landman et al. (2003) have first suggested that it is possible to reorient spatial attention among VWM representations during maintenance by showing no difference in performance between single and sequential attention shifts. However, as noted in the introduction, it is difficult to draw firm conclusions from their data due to the lack of statistical significance. In our experiments, we refined the procedure of Landman et al. (2003) by including neutral and invalid trials. In all three experiments, we found reliable facilitative effects of the second attentional cues, supporting the view of Landman et al. (2003) that the initially uncued items are accessible for later attentional selection. Since there was a significant benefit of the first spatial cue in the SE condition, we can rule out the possibility that participants are able to use the second attentional cue just because they have ignored the first cue.

Matsukura et al. (2007) reported that participants were not able to reorient attention to representations at the initially uncued locations. Note that in their experiments, a spatial cue indicated multiple representations, whereas in our experiments, a spatial cue indicated only one representation. The second cue effects observed in our experiments but no such effects in Matsukura et al.’s (2007) experiments may reflect capacity limitations of attention orienting within VWM. One can argue that as attention can be distributed to about four items simultaneously which is suggested by numerous studies using multiple object tracking tasks (e.g., Pylyshyn and Storm, 1988; Sears and Pylyshyn, 2000; Scholl, 2001; Cavanagh and Alvarez, 2005), it is possible that attention can also operate on multiple VWM representations. We admit this possibility, but in this case, more processing resources may be required, consequently introducing extra interferences toward the maintenance of uncued representations. This can cause failure in reorienting attention. Indeed, Makovski and Jiang (2007) have demonstrated that the facilitative effect of the retro-cue was only observed when a single item was cued, whereas retro-cueing participants to attend multiple representations did not benefit performance. Their results suggest that it is much more difficult and might take extra effort to retrospectively orient attention to multiple representations compared to orienting attention to only one representation.

### INVALID RETRO-CUEING MAY NOT NECESSARILY LEAD TO ACCURACY COSTS

In the two single-shift conditions of Experiments 2 and 3, *d*′ on valid spatial cue trials was significantly higher than neutral trials (first-cued vs. neutral: all *p*s < .001), but *d*′ on invalid spatial cue trials did not differ significantly from neutral trials (uncued vs. neutral: all *p*s > .15). Thus, there were only benefits but no costs in these conditions. These results seem to be inconsistent with previous research that reported both benefits of valid retro-cueing and costs of invalid retro-cueing (Griffin and Nobre, 2003). However, recent research has provided new evidence that invalid retro-cueing does not necessarily lead to accuracy costs, especially when the number of items in the memory arrays is within the VWM capacity limit of about four objects (Astle et al., 2012). For example, Astle et al. reported equivalent *d*′ for invalid and neutral trials with a memory load of four items, but significantly worse *d*′ for invalid relative to neutral trials with a memory load of eight items. Based on these findings, the authors suggested that participants use retro-cues “in a strategic way, only discarding items if it is essential.” In our experiments, the first cue might have been strategically used to facilitate the cued location, but not to suppress the uncued locations, in case a second spatial cue requiring access/retrieval of an uncued representation appeared.

## CONCLUSION

The present study has demonstrated that it is possible and advantageous to reorient spatial attention within VWM, supporting the view that initially unattended representations can be accessed/retrieved by later reorientation of attention (Landman et al., 2003). Our results are consistent with research on object-based reorienting within VWM (Lepsien and Nobre, 2007), and therefore reveal commonalities in different forms of attention reorienting. Moreover, the finding of preserved first cue benefit after attention reorienting indicates that suppression of the previously attended location can be sufficiently controlled. In sum, our findings provide new information on the nature of dynamic attentional control within VWM.

## Conflict of Interest Statement

The authors declare that the research was conducted in the absence of any commercial or financial relationships that could be construed as a potential conflict of interest.
